# Pediatric Obesity Algorithm: A Practical Approach to Obesity Diagnosis and Management

**DOI:** 10.3389/fped.2018.00431

**Published:** 2019-01-23

**Authors:** Suzanne E. Cuda, Marisa Censani

**Affiliations:** ^1^Department of Pediatrics, Baylor College of Medicine, Children's Hospital of San Antonio, San Antonio, TX, United States; ^2^Division of Pediatric Endocrinology, Department of Pediatrics, Weill Cornell Medicine, New York Presbyterian Hospital, New York, NY, United States

**Keywords:** children, obesity, algorithm, adolescents, comorbidities

## Abstract

Childhood obesity is a growing global health problem. Despite the highest rates of childhood obesity in the United States and other developed countries over the last 30 years, there is still no clear treatment strategy. Practitioners often do not know where to turn to find guidance on managing the nearly one third of their population who present for medical care either with obesity that coexists with other medical problems or because of obesity. The Pediatric Obesity Algorithm is an evidence based roadmap for the diagnosis and management of children with obesity. In this article, we summarize topics from the Pediatric Obesity Algorithm pertaining to pediatric obesity diagnosis, evaluation, and management including assessment, differential diagnosis, review of systems, diagnostic work up, physical exam, age specific management, comorbidities, use of medications and surgery, and medication associated weight gain. Identifying and treating children with obesity as early as possible is important, as is identifying comorbid conditions. Earlier and more comprehensive management through resources such as the Pediatric Obesity Algorithm serve to help guide health care practitioners with a practical and evidence based approach to the diagnosis and management of children with obesity, and provide families with the tools needed for a healthy future.

## Background

Childhood obesity is a growing global health problem. Despite a continual rise in the rate of childhood obesity in the United States and other developed countries over the last 30 years, there is still no clear treatment strategy. A great deal of the research effort into solving the problem of childhood obesity is directed toward prevention. There are few evidence based studies specifically addressing the treatment of childhood obesity, thus the management and treatment of the child with obesity is left to the practitioner to use clinical judgment and persuasion to modify the family's dietary and lifestyle habits (1–3).

Often, societal barriers pose roadblocks to early diagnosis and referral for treatment. Parents frequently do not recognize the problem until it is advanced and practitioners are neither adequately trained nor have the clinical support they need to provide the ongoing chronic care needed to manage a child with obesity. Pediatric weight management clinics are spread across the country leaving large swathes of areas where referral to these clinics is not reasonable. Bariatric surgery, while being done more frequently in adolescents, is still reserved for adolescents with severe obesity, and is best accomplished in a center with expertise.

Newer medications have come onto the market for the treatment of adults with obesity, but none of these newer medications are currently FDA approved for use in children with obesity. Endoscopic procedures are also in clinical trials, but adults are the target group. In the meantime, rates of severe obesity continue to increase, especially in minority and low income children (3).

Practitioners often do not know where to turn to find guidance on managing the nearly one third of their population who present for medical care either with obesity that coexists with other medical problems or because of obesity. The Pediatric Obesity Algorithm (4) is an evidence based roadmap for the diagnosis and management of children with obesity. These age specific recommendations are meant to be used by practicing clinicians managing children with obesity. The topics addressed range from assessment to the diagnosis and treatment approach of obesity comorbidities. The Pediatric Obesity Algorithm was created by a collaboration of clinicians from the Obesity Medicine Association who reviewed and summarized the literature and is intended for use by health care providers in clinical practice, research, and education. This manuscript is based on the initial version of the Pediatric Obesity Algorithm, sponsored by the Obesity Medical Association and launched in September 2016 (4).

## Initial Assessment

Epigenetics is a term used to describe processes that result in heritable regulation of gene expression without a change in the base sequence of DNA sequence. Epigenetics is thought to play a large role in the precipitous rise in obesity over the past 30 years. Children are at increased risk for obesity if their parents have obesity: there is a 30% chance of obesity if one parent has obesity and a 90% chance if both parents have obesity. Obesity in childhood is associated with a maternal preconception BMI (body mass index) ≥30 kg/m^2^, excessive gestational weight gain, and gestational diabetes mellitus (5–7). Infants who are small for gestational age due to tobacco abuse or insufficient maternal weight gain are also at risk for obesity and metabolic disease in childhood. Other exposures that can result in obesogenic epigenetic changes include but not are limited to: toxins, nutrition, medications, antibiotics, infection, and exogenous hormones (8–10).

Weight assessment of a child with obesity is accomplished by considering both the age of the child and the severity of the obesity. For infants up to the age of 2, BMI is not assessed. Instead, the infants' weight percentile is compared to length percentile. There are two options for the use of growth charts in infants up to the age of 2 years: Center for Disease Control (CDC) charts which are based on a cohort of mostly Caucasian American infants who were mostly bottle fed or WHO charts which are based on infants from multiple areas of the planet with diverse racial and ethnic backgrounds who were mostly breastfed. An infant whose weight for length percentile is increasing, or “jumping” percentile lines needs closer monitoring than one who is maintaining growth along the same percentile.

Body mass index charts are used for children between the ages of 2–20 years. These CDC charts were developed in 2000 and are color coded by BMI percentile: < 5th percentile (red), 5th−85th percentile (green), 85th−95th percentile (yellow), and >95th percentile (red). The chart extends to a BMI of 35 kg/m^2^. These charts were developed using five cross sectional nationally representative health surveys taken between the years of 1963–1994 (11). An additional BMI chart has been developed for children aged 2–20 years with severe obesity (12). On this chart, percentile lines are included for BMI measurements between 110 and 190% of the CDC 95th percentile. Both of these BMI charts display a “J” shaped curve with the BMI of young children decreasing normally between the ages of 2 and 6 years and then steadily increasing between the ages of 6–20 years. This decrease or dip and subsequent rise in the BMI curve is referred to as adiposity rebound. If a child's BMI either has no decrease or prematurely rises between the ages of 2 and 6 years, the child is at risk or has obesity. This phenomenon is called early adiposity rebound.

In June 2012 the American Medical Association declared obesity a disease. Children, like adults, suffer from the manifestations of obesity on most aspects of their physical and psychological health. Adiposopathy is a term used to describe endocrine and immune responses to increased adipose tissue while fat mass disease describes the physical response to increased adipose tissue (13, 14). A careful history that includes family history, prenatal, birth and postnatal care, followed by any medical complications in childhood and medications used both for the management of comorbid conditions and the management of obesity should be obtained.

**Figure 1:**
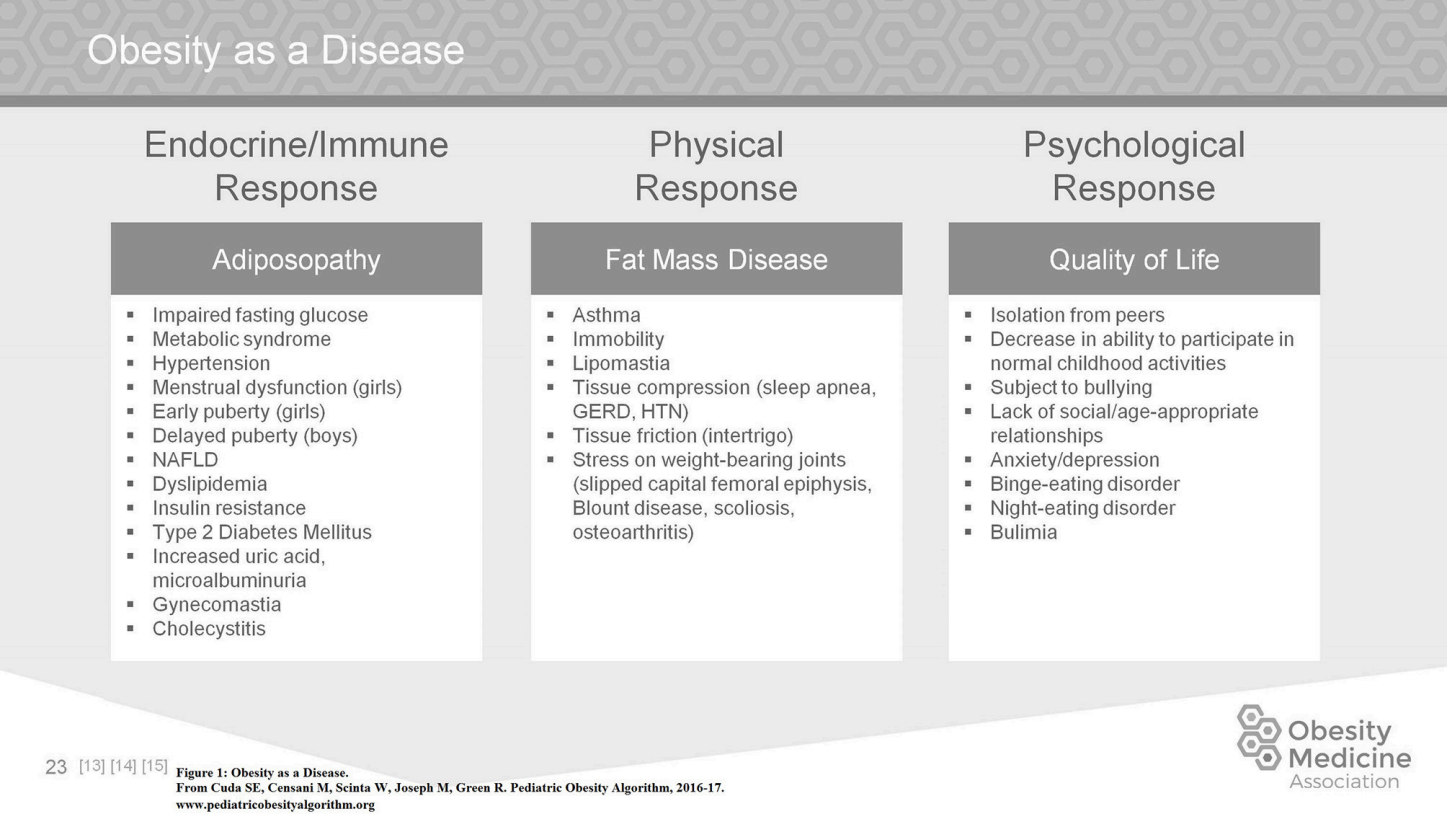
Obesity as a disease.

In addition, psychological issues arise as a result of obesity and affect quality of life (Figure 1). The quality of life of children with obesity may be poor. They are at increased risk for isolation from peers, they are subject to bullying, they are at increased risk for anxiety and depression, and they are at increased risk for eating disorders, especially binge eating disorder, night eating disorder, and bulimia (15–17).

Social history includes not only a dietary recall, but also a history of breast or bottle feeding, the timing of introduction of complementary foods and parenting style. In addition, an assessment of the child's activity level including access to safe areas to exercise and support for a high level of activity is important. Finally, the clinician needs to assess sedentary time and non-academic screen time.

The differential diagnosis of children with obesity starts with an assessment of linear growth. Linear growth proceeds in children until fusion of growth plates. Children with obesity due to nutritional, also referred to as endogenous, obesity have consistent or accelerated growth. These children are at risk for early development of secondary sexual characteristics and may have bone age development that exceeds their chronological age by more than 2 standard deviations. In contrast, a child with obesity who has an underlying endocrinopathy will typically have decreased linear growth. It is in the children with a deceleration in growth that testing for thyroid hormones is indicated. If there is clinical suspicion of Cushing's syndrome, a dexamethasone suppression test or 24 h urinary free cortisol level is indicated (1, 2, 14).

Genetic causes of obesity should be considered in children with severe obesity before the age of 5 years. These young children may present with developmental delay, short stature, dysmorphic facies, or hyperphagia. Screening involves DNA methylation studies, exome sequencing and karyotyping looking for known syndromes: Prader Willi, Bardet-Biedl, Fragile X, Algrights Hereditary Osteodystrophy, Alstrom, Congenital Leptin deficiency, POMC deficiency, MC4R deficiency, and Cohen syndrome. However, many other genetic causes of obesity not associated with known syndromes no doubt contribute as well (18–20).

Children with special needs are at increased risk of developing obesity (21). Some of these children are at increased risk due to associated difficulties with movement or coordination. Developmentally delayed or special needs children can present with decreased or normal growth. Presentation is highly variable and the practitioner should take a careful family history and consider a referral to a geneticist.

**Figure 2:**
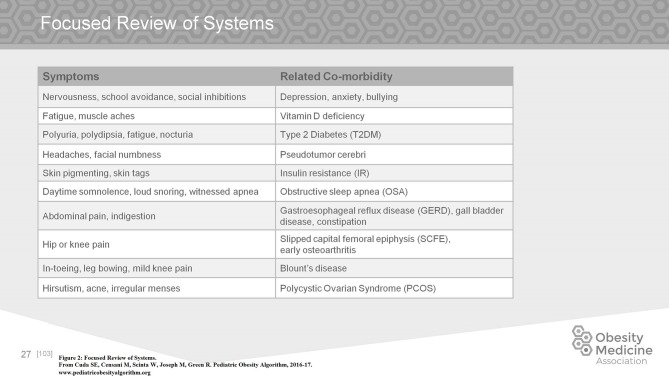
Focused review of systems.

Clinical evaluation of the child with obesity includes a focused review of systems. Figure 2 lists the more commonly presenting signs or symptoms and their related comorbidities (22).

**Figure 3:**
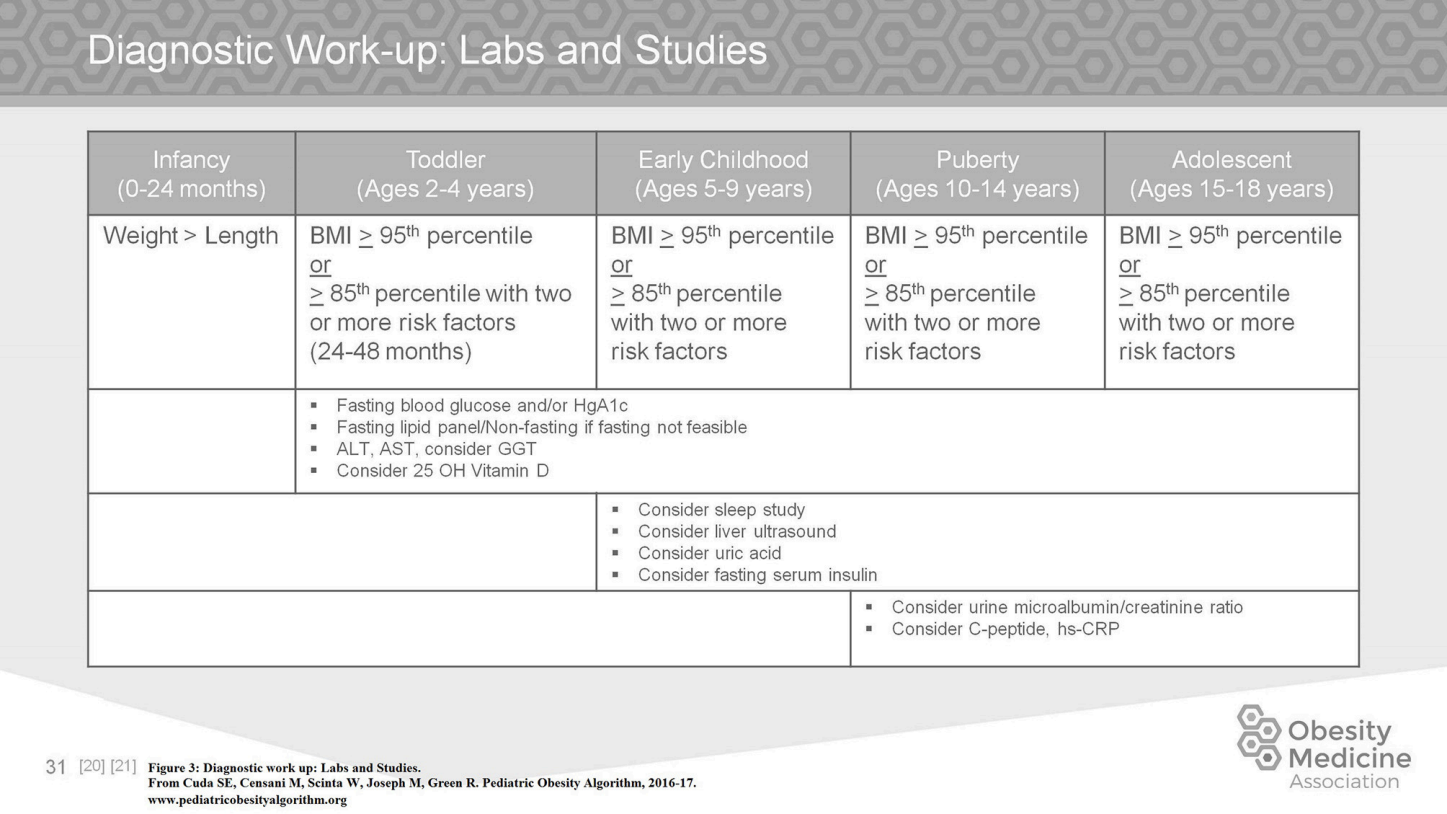
Diagnostic work up: labs and studies.

The diagnostic work up of a child with obesity is driven by a careful history of prenatal factors, family history, feeding history, sleep duration and issues, exercise, family and cultural expectations, screen time, location and timing of meals, bullying or social isolation, motivation and ability to make modifications of the family, and finally financial constraints. Appropriate labs and studies are considered according to the age, BMI percentile, and presence of risk factors as summarized in Figure 3 (1–3, 23, 24).

Children with obesity presenting with deceleration of growth, symptoms of hypo or hyper thyroidism or other endocrinopathies, symptoms of diabetes, sustained hypertension, hirsutism, a family history of early cardiovascular disease, snoring, and/or daytime sleepiness will need additional workup. In addition, there are many other complications of obesity that require further investigation, some of which are discussed in the section on comorbidities.

## Approach to Physical Exam

The physical exam is both important and challenging in children with obesity. While increased adiposity is usually apparent, children may go to considerable effort to conceal problems, for example removal of excess hair. Children with obesity commonly present wearing more clothing than called for by climatic conditions and may be wearing spandex or other restraining garments under their loose outerwear. The practitioner should take particular care to preserve the child or adolescent with obesity's need to cover up while still examining the patient. Instead of asking the child or adolescent to fully unclothe, the practitioner can examine the patient sequentially, taking care to reclothe the body parts that are exposed before moving on to the next body part. A thorough discussion of all of the physical findings that can be associated with obesity does not follow, however we highlight a few areas that should be assessed in every child.

Acanthosis nigricans is a cutaneous marker associated with hyperinsulinemia which is frequently perceived by the parents or the child as being due to dirt or eczema, not melanin. An explanation of the cause can be reassuring to the child and the parent and provide opportunity for education of the physiology of glucose metabolism and underlying process of insulin resistance.

Pubertal or tanner staging helps the clinician determine growth potential as well as address the issue of premature thelarche in females or gynecomastia in males. These findings are exacerbated by excess adiposity. Pubertal status also informs laboratory results as some normal values change as puberty progresses.

The clinician should be aware of the skeletal problems that occur in children with obesity. Young children with the bowed tibias of Blount's disease are usually ambulatory and may be “early walkers” and unaware of any problem. A radiologic diagnosis is necessary. Slipped capital femoral epiphysis can present as knee or hip pain or without pain resulting in the diagnosis being missed. Prompt assessment and referral to an orthopedic surgeon if the diagnosis is made is necessary. Scoliosis is harder to detect due to adiposity despite occurring in children with obesity at as great or greater a frequency than in normal weight children (25–27). The practitioner should have a high index of suspicion for physical abnormalities and should carefully examine the child. Children with obesity are frequently poorly evaluated by the medical community until symptoms become severe.

A careful examination of the entire body for intertrigo, especially if this complaint is inhibiting the child's activity level should be performed. A sensitive examination of the status of excess hair in females should occur. Other findings from the review of systems may determine the examination: for example a history of snoring should prompt a thorough exam of the tonsillar pillars as well as neck circumference.

## Management of Obesity

**Figure 4:**
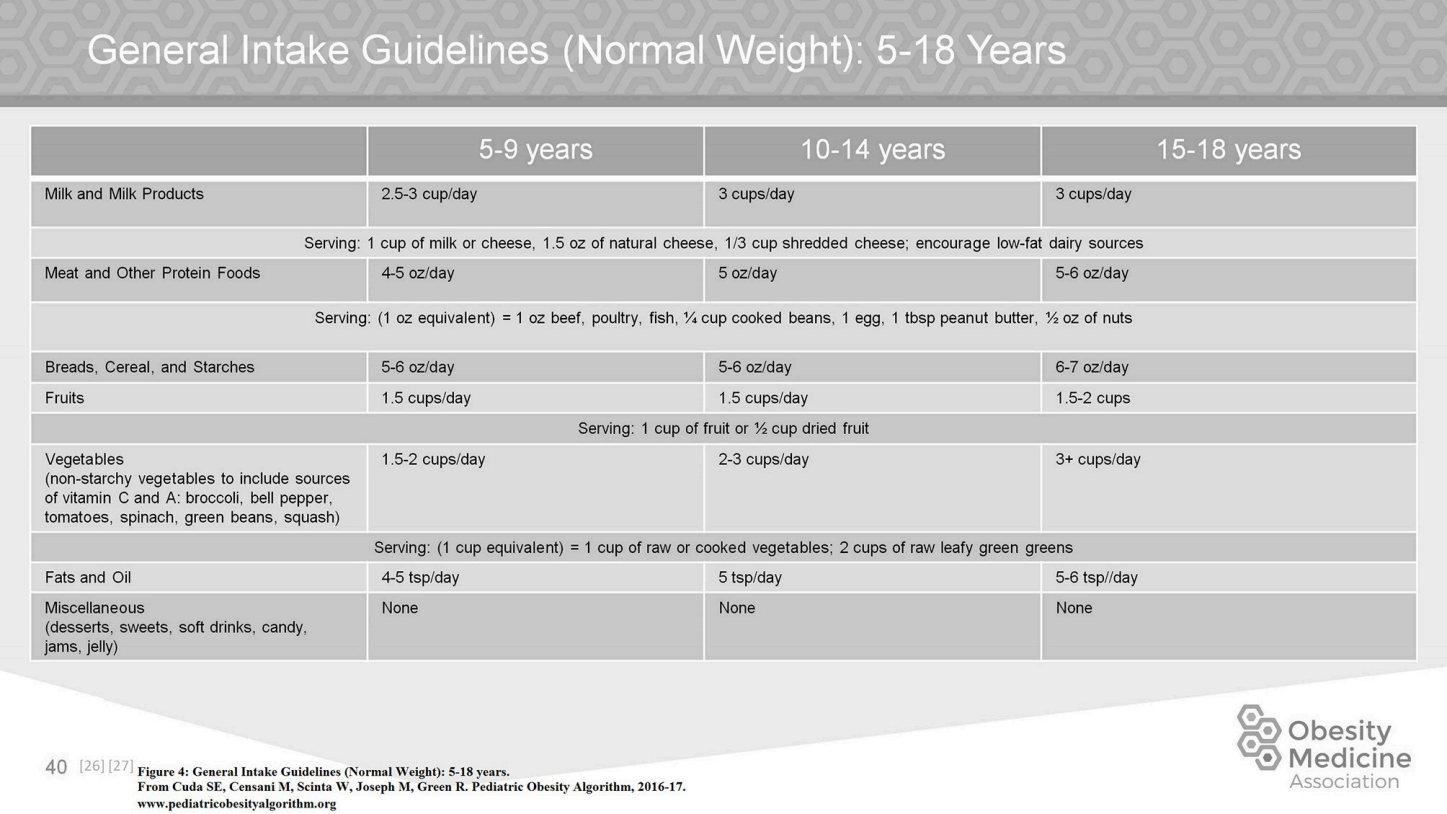
General intake guidelines (normal weight): 5–18 years.

In considering how to modify the food intake of a child with obesity, there is no universally accepted approach. An understanding of appropriate intake for a normal weight child is necessary as a starting point. For easy reference, the algorithm breaks down intake guidelines for age groups between infancy and adulthood (Figure 4).

**Figure 5:**
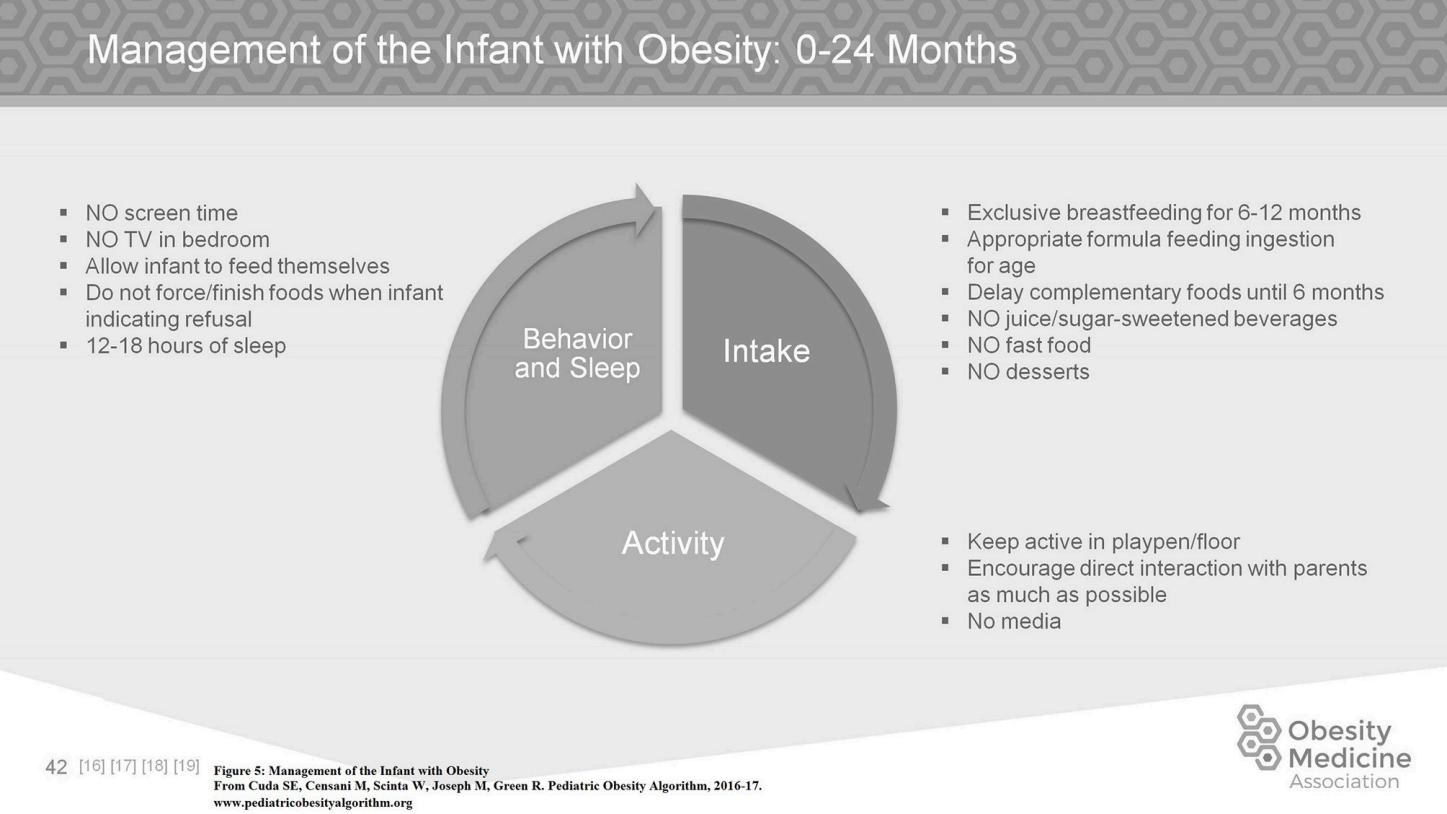
Management of the infant with obesity.

Managing a child with obesity is age dependent. In the first 6 months of life, exclusive breast feeding is the nutrition of choice. Complementary foods should ideally be delayed until 6 months of age. Increased BMI in childhood and adolescence is associated with early introduction of complementary foods. Infants with obesity should not be given any sugar sweetened beverages, nor any fast food or desserts. Infants already struggling to maintain their weight should have age appropriate amounts of formula, and should not be given juice in their bottles. Infants should not be watching TV or any screen of any kind for the first two years of life. Normal infants may need to sleep up to 18 h a day, and should sleep at least 12 h a day. Infants should be allowed to be as active as possible, either on the floor or in a playpen and the parents should be encouraged to have as much direct interaction with them as possible (Figure 5).

A toddler (age 2–4 years) with obesity should have three meals plus 1–2 snacks every day. They should not be offered sugar sweetened beverages, nor any fast food. Portion sizes should be age appropriate and they should be praised for trying new foods. Parents should model the eating behavior they want their child to have. Toddlers should have a routine sleep pattern. Snoring in this age group is frequently associated with tonsillar hypertrophy. If the tonsils cause significant obstruction, removal may be indicated.

Up to the age of 2 years, no screen time is recommended. Between the ages of 2 and 4, screen time should be kept to a minimum. Obesity is directly correlated with screen time in this age group. The family should adopt good meal hygiene to include meals at the table, no media while eating, no food rewards, no over controlling behaviors toward consumption of meals, and family based meals.

Obesogenic medications may be a factor for the young child with obesity (aged 5–9 years). The use of second generation antipsychotics should be minimized and asthma should be managed with controller medications instead of systemic steroids if clinically possible. Children at this age may also develop hypertension as a complication of their obesity, however etiologies other than obesity must be considered.

Parents are strong role models for children at this age and involvement of the family in the care of the child with obesity is highly recommended. Total non-academic screen time should be kept to a minimum. Replacement of screen time with moderately vigorous physical activity is associated with a decrease in obesity. Sleep is still very important with children in this age group needing between 11 and 14 h of sleep, preferably all at once and not achieved by napping during the day due to deficits at night.

**Figure 6:**
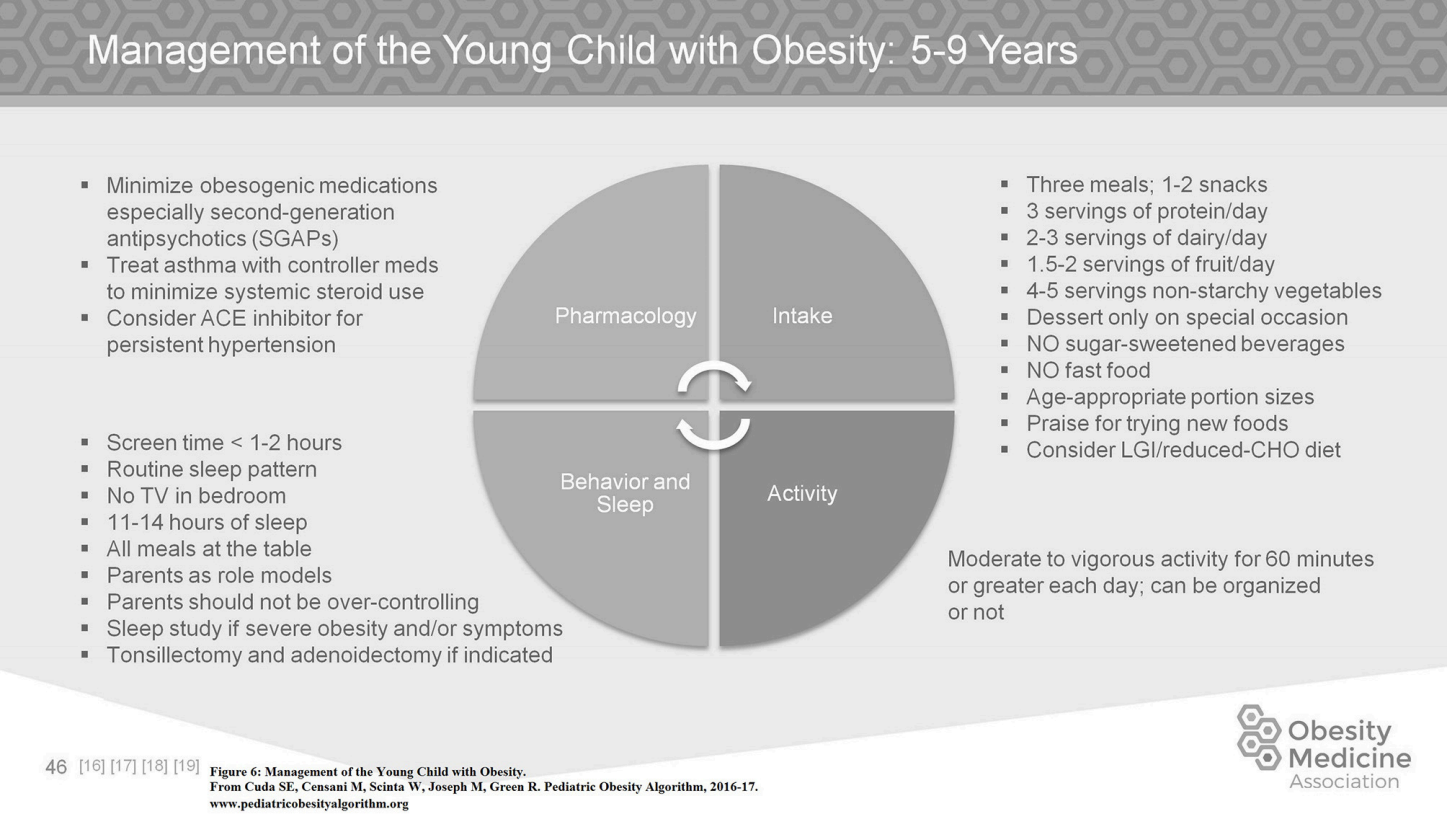
Management of the young child with obesity.

Children with obesity in the 5–9 age group should be consuming 3 meals per day plus 1–2 nutritious snacks. Food groups should include 3 servings of protein per day, 1–2 servings of dairy per day, and 4–5 servings of non-starchy vegetables per day. They should not be consuming any sugar sweetened beverages, nor any fast food. Portion sizes should be age appropriate and children should be praised for trying new foods (Figure 6).

Children 5–9 years of age start to be involved in organized sports as well as continuing to need active play. Activity should be daily, as vigorous as possible, but fun. Sixty minutes or more per day of moderate to vigorous physical activity is recommended. Children ages 5–9 with obesity should be encouraged to exercise as often and as vigorously as normal weight children. Limitations due to adiposity are rare in this age group.

As children with obesity go through puberty and adolescence, management evolves. Many of the same recommendations as made in younger children are still valid, although must be adapted to the older child. In particular, meals should continue to be consumed at 3 a day and can include 1–2 snacks. Older children and adolescents with obesity frequently skip meals leading to overindulgence at the next meal or eating the majority of their intake starting in the afternoons and into evenings. Techniques for dealing with the almost constant exposure to non-nutritious foods should be discussed with the older child and adolescent since they are increasingly consuming food outside of the family home and school.

**Figure 7:**
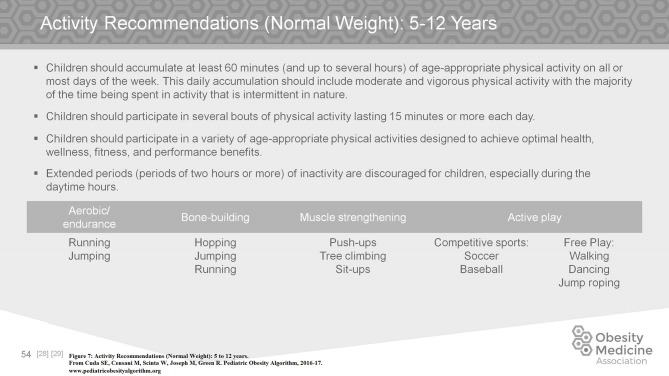
Activity recommendations (normal weight): 5 to 12 years.

Older children and adolescents with obesity are able to track their exercise and meal intake using newer technologies which allow them to share their progress and compare themselves to their peer groups. In the age of the smart phone, children in this age group are rarely without their devices. These devices function as convenient activity trackers (28). After puberty, adolescents become less active in general. However, for the adolescent with obesity it is important to develop a regular exercise routine of 60–90 min of moderate to vigorous activity per day (Figure 7). This increased activity will preserve or increase cardiorespiratory fitness which mediates the development of Type II diabetes mellitus and/or the metabolic syndrome (29–31). A gradual increase in activity in those who are starting from relative inactivity is suggested.

Duration and quality of sleep should be addressed. Adolescents in particular may reverse their sleep wake cycles by staying up late at night, usually on social media or video games, and then sleep until midday or later. Correction of the sleep wake cycle is necessary to allow for a normal pattern of eating, especially eating with other family members. Electronic devices should be removed from bedrooms to allow uninterrupted sleep. Inadequate sleep contributes to hunger. Adolescents frequently need up to 10 h or more of sleep per night (32, 33).

## Obesity Comorbidities

Comorbidity management complicates the treatment of children with obesity. Although children with obesity can present with all the same disease processes seen in normal weight children, certain disease processes occur secondary to obesity. The significance of these disease processes cannot be understated as they progress through adulthood and are associated with premature morbidity.

Hypertension occurs with an increased incidence in children with obesity as compared to normal weight children. The diagnosis of hypertension must be based on three separate measurements at least 1 week apart. Guidelines for diagnosis of hypertension in children now differ between children less than or greater than the age of 13. After the age of 13, a systolic blood pressure of 120–129 mm Hg and a diastolic pressure < 80 mm Hg is considered elevated, while pressure of 130–139 mm Hg over 80–89 mm Hg are Stage I hypertension, and >140/90 mm Hg is Stage II hypertension. For children < 13, diagnosis should be made by referring to normative date based on age, sex, and height. A diagnosis of stage II hypertension in a child or adolescent with obesity should include as assessment of end organ damage. Diagnostic studies may include a renal Doppler ultrasound, ECHO, serum uric acid, urine protein, and BUN/creatinine levels. Treatment should include a trial diet and lifestyle modifications prior to use of medication. A low sodium (< 1,500 mg/day) or DASH (Dietary Approaches to Stop Hypertension) diet is usually recommended. Pharmacotherapy is used if the blood pressure is persistently elevated over 3 separate measurements and does not respond to lifestyle intervention. The primary treatment for obesity associated hypertension is weight loss (34–37).

Dyslipidemia is commonly found in children with obesity. Typically, the dyslipidemia of obesity is a high triglyceride level and low high density lipoprotein (HDL) level. This pattern usually quickly responds to dietary modification. The clinician should be aware that children with obesity can present with any dyslipidemia that occurs in normal weight children. If the pattern of dyslipidemia is different than an elevated triglyceride level and/or low HDL level, work up and treatment is detailed in the National Heart, Lung, and Blood Institute guidelines. On initial presentation, a child with obesity who consumes a large amount of sugar sweetened beverages can present with a triglyceride level between 150 and 400 mg/dl. Removing the refined carbohydrates from the child's diet will usually have a profound effect on the triglyceride level. If there is no response, further evaluation is needed (38, 39).

Sleep Disorders in children with obesity can have varied presentations including apnea associated with snoring or disrupted sleeping, daytime sleepiness, hyperactivity, depression, audible pauses in breathing, new onset nocturnal enuresis, irritability, and learning difficulties. The clinician's index of suspicion must be high as sleep disorders can also occur in the absence of symptoms. Referral for sleep studies or possible removal of tonsils and/or adenoids is based on clinical evaluation (40–42).

Impairment in glucose metabolism is important to screen for in children with obesity. Impaired fasting glucose is defined as a fasting plasma glucose of ≥100 mg/dl but < 126 mg/dl on repeat measurements. Impaired glucose tolerance is defined as a 2 h plasma glucose on oral glucose tolerance test (OGTT) of ≥140 mg/dl but < 200 mg/dl. Type 2 diabetes mellitus is the endpoint of metabolic decompensation that may evolve over months to years. In insulin resistance, the body produces insulin but muscle, fat, and liver do not respond properly to insulin and thus cannot easily absorb glucose from the bloodstream, with progression from insulin resistance to prediabetes with impaired glucose tolerance and impaired fasting glucose over time (39). Clinical findings of insulin resistance on physical exam include acanthosis nigricans, skin tags, and hyperpigmentation in axillae, umbilicus, groin, and popliteal fossae. Insulin resistance is also associated with polycystic ovarian syndrome. Treatment is weight loss through aggressive diet and lifestyle intervention including modified carbohydrate diets and low glycemic index food choices (43–47). Metformin is an insulin sensitizer that is FDA approved in children ≥10 years of age for the treatment of type 2 diabetes mellitus. Metformin decreases hepatic glucose production, decreases glucose intestinal absorption, and improves insulin sensitivity through increasing peripheral glucose use and uptake (48, 49).

**Figure 8:**
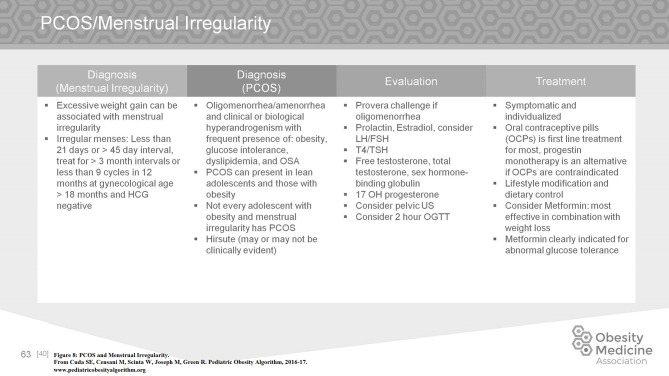
PCOS and menstrual irregularity.

Excessive weight gain can be associated with menstrual irregularity. Irregular menses is defined in adolescence as < 21 days or >45 day intervals or < 9 cycles in 12 months at gynecological age >18 months, and cycles < 3 days or >7 days duration. Polycystic ovary syndrome (PCOS) is one of the most common endocrine disorders affecting young women seen with oligomenorrhea or amenorrhea and clinical or biological hyperandrogenism with frequent presence of obesity, glucose intolerance, dyslipidemia, and obstructive sleep apnea. PCOS can present in lean adolescents as well as adolescents with obesity. In the evaluation of irregular menstrual cycles, hormonal evaluation is indicated including free testosterone, total testosterone, early morning 17 OH progesterone to rule out late onset congenital adrenal hyperplasia, DHEA-S, sex hormone binding globulin, thyroid function tests, and prolactin as well as consideration for pelvic ultrasound and a 2 h OGTT. Treatment is symptomatic and individualized. Oral contraceptive pills (OCPs) are first line treatment for most adolescent patients to help to regulate menstrual cycles and to lower the level of testosterone to improve acne and hirsutism. Progestin monotherapy is an alternative if OCPs are contraindicated. Treatment includes lifestyle modification and dietary control with studies finding metformin therapy when indicated to be effective in combination with weight loss (50, 51). Additional treatments such as antiandrogen therapy may be considered but are outside the scope of the Algorithm. An example of the approach to diagnosis, evaluation, and management of obesity comorbidities in the Pediatric Obesity Algorithm is found in Figure 8 pertaining to PCOS.

Vitamin D is a fat soluble vitamin essential for skeletal health in growing children. It plays an important role for bone health through the absorption of calcium from small intestine, and is available in diet and through synthesis from sunlight. Vitamin D deficiency has been defined by the Institute of Medicine and Endocrine Society clinical practice guidelines as a serum 25-hydroxyvitamin D [25(OH)D] < 20 ng/mL. Current recommendations for children aged 1–18 years include treatment with 2,000 IU daily of vitamin D2 or vitamin D3 for at least 6 weeks or 50,000 IU of vitamin D2 or D3 once a week for at least 6 weeks to achieve a blood level of 25(OH)D above the deficient range, followed by maintenance therapy of 600–1,000 IU daily. There should be special considerations for at risk patient populations including children with obesity, malabsorptive syndromes, or on medications affecting vitamin D metabolism such as anticonvulsants, glucocorticoids, antifungals, and antiretrovirals. Lower levels of vitamin D in obesity have been linked to decreased sun exposure and reduced dietary intake, in addition to decreased vitamin D bioavailability secondary to storage of fat soluble vitamin D in adipose tissue. These children may require 2 to 3 times the dose of vitamin D to achieve the same serum 25(OH)D levels as children without these conditions (52–56).

## Medications and Surgical Approach

Bariatric surgery is reserved as treatment for severe obesity in adolescents. The number of surgeries performed is increasing each year. Surgical options include gastric sleeve resection, Roux-en-y gastric bypass, and laparoscopic adjustable gastric banding under the treatment of an experienced surgical center. Current adolescent bariatric recommendations include BMI >35 kg/m2 with moderate to severe comorbidities or BMI >40 kg/m2 and skeletal and sexual maturity (generally age 14 for girls and 15 for boys). Participation in a weight loss program with a multidisciplinary team involving pediatric surgery, nutrition, psychology, and pediatric specialists is recommended. Females are counseled on increased fertility and all adolescents give informed consent. Significant psychiatric disease is considered an exclusion criteria. There are limited data outcomes; however, one 3 year outcome study found mean percent weight loss of 27%, normalized blood pressure in 74%, normalized lipid levels in 66%, and over 50% of patients with T2DM in remission (57–61).

**Figure 9:**
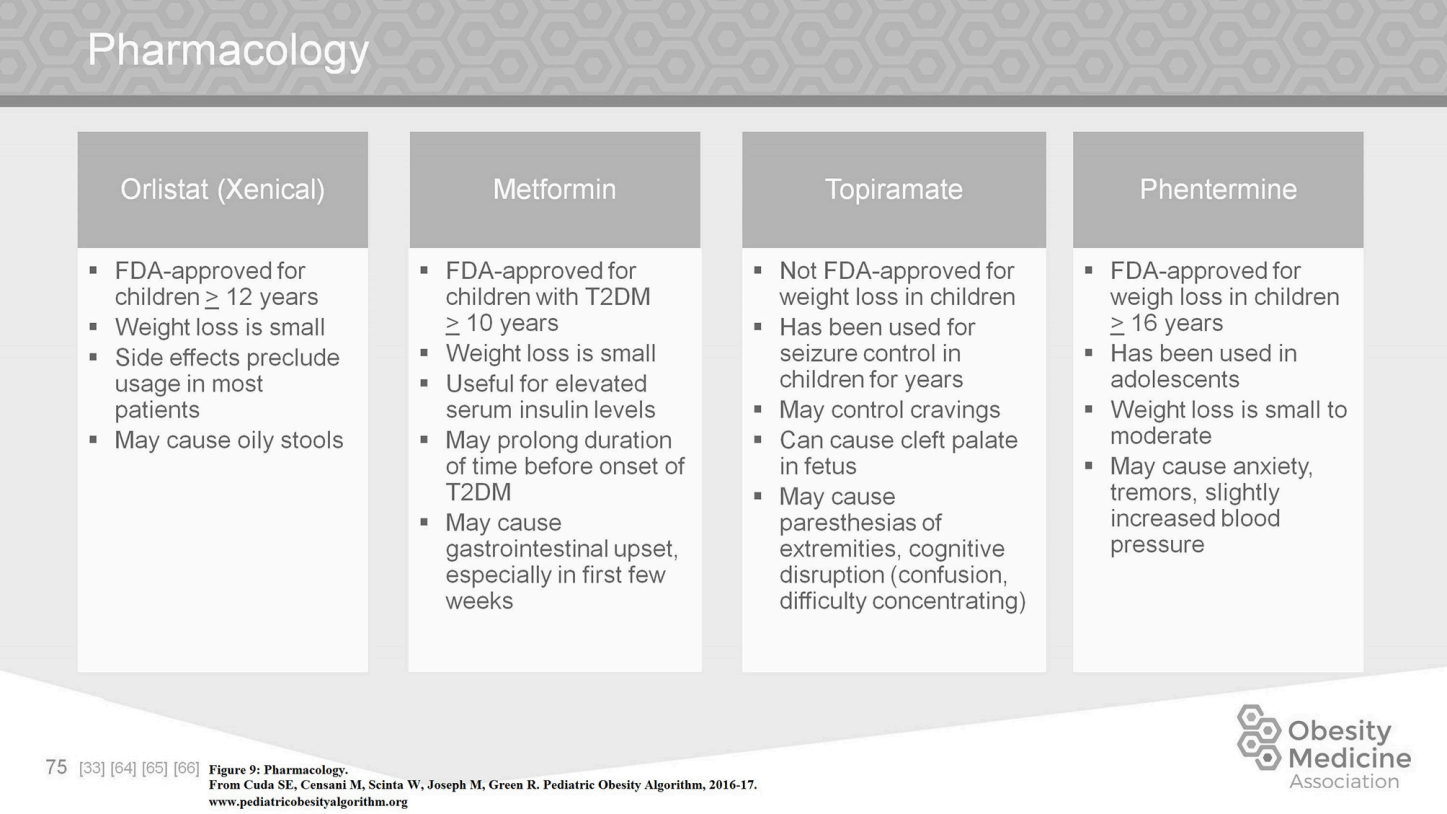
Pharmacology.

The use of weight loss medications in children with obesity is limited. Although there are several new medications on the market for adults, none have been FDA approved for children. Of the approved medications, orlistat can produce a small amount of weight loss but is associated with oily stools, a side effect not tolerated by many children. Topiramate is used in children as an antiepileptic medication but is not FDA approved in children for weight loss. Although the mechanism is unclear, it can help control cravings. However, topiramate must be used with caution due to side effects of paresthesias of the extremities, and cognitive disruption, especially at higher doses. In addition, it can cause cleft palate in the fetus, making it more complicated to use in an adolescent female. Phentermine is approved in children >16 years of age for weight loss. The associated weight loss is small to moderate (62–68). Phentermine may cause anxiety, tremors, and slightly increased blood pressure (Figure 9).

**Figure 10:**
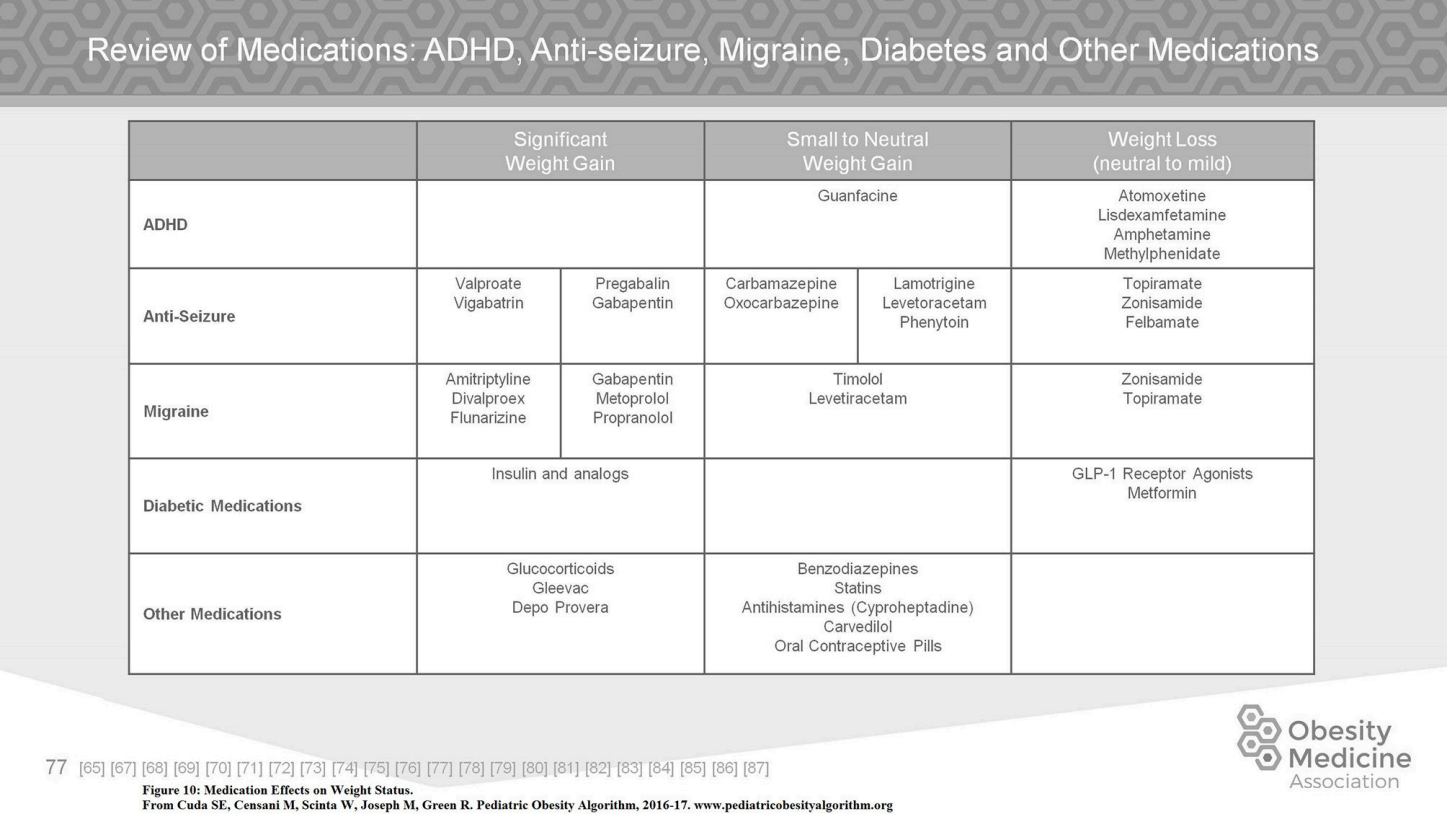
Medication effects on weight status.

**Figure 11:**
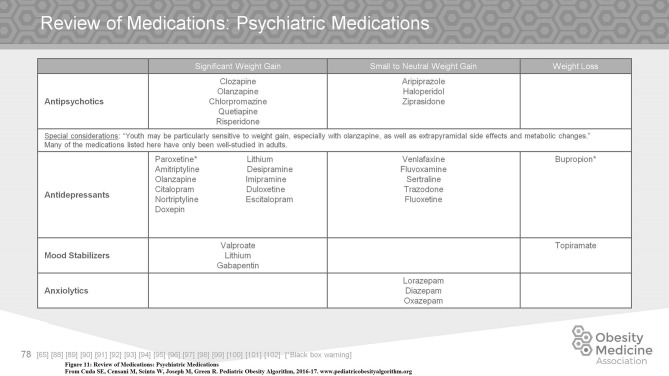
Review of medications: psychiatric medications.

Medication associated weight gain is a significant management issue. The use of second generation antipsychotics, most of which are associated with weight gain, has increased markedly over the past decade. Many other commonly used medications in children include but are not limited to antidepressants, anxiolytics, mood stabilizers, antiseizure medications, migraine medications, antihypertensives, diabetic medications, glucocorticoids, and progestins. On assessing a child with obesity on medications, the clinician must first work within the pathology of the underlying problem. If possible, a substitution for a less obesogenic medication may aid in controlling weight gain. Metformin is frequently used to offset weight gain secondary to psychiatric medication. Controversy concerning the efficacy of the use of Metformin for this purpose exists (69, 70). Figures 10, 11 summarize commonly used medications for children and their effects on weight.

## Conclusion

Obesity is a chronic disease which when originating in childhood can lead to medical and psychological complications and premature comorbidity and mortality. An increasing amount of children with obesity presenting for treatment have BMIs well above the 95th percentile and the increased amount of children with severe obesity concerns most clinicians. Identifying and classifying these children as early as possible is important, as is identifying comorbid conditions. Frequently the management of a child with obesity is not just to decrease their BMI but to minimize their disease state and change their development of further complications. A slowing of weight gain or lack of continued acceleration of weight gain can delay the onset of T2DM and early cardiovascular disease. In fact, addressing comorbid conditions such as obstructive sleep apnea, behavioral disorders, polycystic ovarian syndrome may be a necessary precursor to successful weight management. Clearly more needs to be done in our attempt to stop and eventually reverse this epidemic. Medical management of children with obesity is conservative based not only on the lack of evidence based studies but also on societal mores and cultural sensitivities. However, the epidemic of childhood obesity is permeating most other medical problems and it is increasingly clear that our current approach to managing obesity in children is not aggressive enough. Earlier and more comprehensive management through resources such as the Pediatric Obesity Algorithm serve to help guide health care practitioners with an evidence based roadmap for the diagnosis and management of children with obesity, and provide families with the tools needed for a healthy future.

## Author Contributions

SC and MC contributed to the research, writing and editing of the manuscript, approved the final version, and agreed to be accountable for the content of this work.

### Conflict of Interest Statement

The authors declare that the research was conducted in the absence of any commercial or financial relationships that could be construed as a potential conflict of interest.
